# Women’s and men’s experiences with participative decision-making at workplace and organizational levels

**DOI:** 10.3389/fpsyg.2023.1240117

**Published:** 2024-02-01

**Authors:** Clara Plückelmann, Marie Gustafsson Sendén, Claudia Bernhard-Oettel, Constanze Leineweber, Sabine Sczesny

**Affiliations:** ^1^Department of Psychology, Stockholm University, Stockholm, Sweden; ^2^Department of Psychology, University of Bern, Bern, Switzerland

**Keywords:** participative decision-making, gender equality, leadership, gender stereotypes, gender roles

## Abstract

**Introduction:**

The concept of participative decision-making (PDM) has been well established as a positive organizational factor, and has recently gained attention as a measure of gender inclusivity in the workplace. However, findings regarding gender differences in the experiences of PDM are inconclusive. This study hypothesized that women perceive themselves as less influential than men at the organizational level rather than at the workplace level. Furthermore, the study explored whether these assumed gender differences depend on the gender typicality of occupational positions and professions. We expected gender differences to be more pronounced for male-typed positions and professions (e.g., leadership, engineer) compared to non-male-typed occupational positions and professions (e.g., non-leadership, nurse).

**Methods:**

Data on experiences with participative decision-making at the workplace and organizational levels were drawn from a large representative Swedish survey (*N* = 10,500; 60% women).

**Results:**

Results showed that women experienced being less influential than men at the organizational level, whereas the experiences of women and men did not differ at the workplace level. The gender difference at the organizational level was not related to the gender typicality of position and profession.

**Discussion:**

The findings highlight the importance of the inclusion of both women and men in strategic, large-scale decisions for achieving gender equality at work.

## Introduction

Participative decision-making (PDM) refers to “the opportunity for an employee to provide input into the decision-making process related to work matters […] or organizational issues” (Valverde-Moreno et al., 2021b). The link between perceived PDM and positive workplace outcomes is well-established. As such, PDM is associated with increased job performance (Sukirno and Siengthai, 2011), self-efficacy (Behravesh et al., 2021), job satisfaction (Pacheco and Webber, 2016), and decreased intent to quit and actual turnover (Spector, 1986). For decades, research has focused on PDM as a beneficial organizational factor (Lowin, 1968; Harrison, 1985; Black and Gregersen, 1997); however, PDM has only recently gained attention as a matter of gender (in)equality in organizations (Shaed and Ishak, 2015; Valverde-Moreno et al., 2019; Mooney, 2022). Women’s and men’s equal inclusion in decision-making at all levels of their organization represents a main indicator of a gender-inclusive climate (Nishii, 2013; Kossek et al., 2017). As such, PDM has the potential to be used to measure gender inclusivity within organizations.

Researchers have proposed that traditional gender roles and gender-based stereotypes contribute to differences between women’s and men’s inclusion in and their experiences of organizational decision-making (Acar Erdol, 2018; Mooney, 2022). Gender stereotypes are widely shared beliefs about women’s and men’s typical characteristics and traits (e.g., Eagly et al., 2020). The content of gender stereotypes traditionally describes women as more communal (e.g., relationship-oriented, social, emotionally sensitive) and men as more agentic (e.g., assertive, bold, dominant; Haines et al., 2016; Eagly et al., 2020). Furthermore, agency encompasses traits that are associated with decision-making competence such as decisiveness and leadership competence (Caleo and Heilman, 2014; Eagly et al., 2020). Additionally, gender stereotypes contain status beliefs that attribute greater status and worthiness to men compared to women in interpersonal settings, including organizations and workplaces (Ridgeway, 2001). Due to the perception that women are less competent decision-makers (Tabassum and Nayak, 2021) and of lower status (Ridgeway and Markus, 2022), men may be given more influence and asked to contribute more often than women (Ridgeway, 2001; Ridgeway and Bourg, 2004). Indeed, CEOs report including female executives less frequently in decision-making than male executives at the same level (Mooney, 2022). Correspondingly, women may experience being less involved in PDM than men (Valverde-Moreno et al., 2019).

So far, past research has revealed inconsistent findings about women’s and men’s PDM experiences. Some studies have found that women reported less PDM than men (e.g., Pacheco and Webber, 2016; Valverde-Moreno et al., 2019), whereas others found that women reported slightly more PDM (Valverde-Moreno et al., 2021b) or did not observe gender differences in PDM (Kahnweiler and Thompson, 2000; Li and Qian, 2016). These inconsistencies may be explained by the level at which PDM takes place, namely the workplace or organizational level. Moreover, women’s and men’s experiences of PDM might be influenced by the gender-typicality of their occupational role, that is, whether they work in a male-typed profession, for instance, as engineers and/or occupy a male-typed status position (being a leader). The aim of the present study was therefore to examine women’s and men’s experiences with PDM at workplace and organizational levels as well as the role of male-typed occupational roles for gender differences in PDM experiences. A comprehensive understanding of women’s and men’s experiences of PDM is important because of its indicative value of gender inclusion in decision-making (Kossek et al., 2017) and its predictive value of positive workplace outcomes (Pacheco and Webber, 2016).

### Gender differences in experienced PDM at workplace and organizational levels

Decision-making at the workplace and the organizational level differs in scope and impact. At the workplace level, decision-making focuses on employees’ immediate tasks and decision-making in smaller groups with less far-reaching consequences and less relevance for the majority of employees of the organization. Such decisions regarding work are referred to as task discretion (Inanc et al., 2015; Valverde-Moreno et al., 2019) or operational participation (Valverde-Moreno et al., 2021b). Examples of workplace level decision-making include: employees’ being able to indicate the order of their tasks (Valverde-Moreno et al., 2019, 2021b) or highlight their training needs (Kahnweiler and Thompson, 2000).

In contrast, at the organizational level decision-making has more far-reaching consequences, which are of relevance for larger groups of employees. Such decisions regarding organizational visions, goals, and missions have been referred to as strategic (Mooney, 2022) or organizational participation (Inanc et al., 2015; Valverde-Moreno et al., 2019, 2021b). Examples of organizational decision-making include: employees being consulted before the setting of objectives (Valverde-Moreno et al., 2019, 2021b); or being included in decision-making regarding policies and rules (Kahnweiler and Thompson, 2000).

When comparing employee participation across the European Union, Sweden (along with Denmark and Finland), exhibited the highest level of involvement compared to other groups of countries. Swedish employees have traditionally been granted high participation in decision-making (Alm and Stern, 2022). In Sweden, the prevailing organizational form endorsed employee participation in decision-making processes, and employees had the opportunity to exert influence not only over their immediate work tasks but also in shaping broader organizational decisions (Gallie and Zhou, 2013). Although employee participation generally is high in Sweden, other aspects of the country’s labor market suggest that the distribution of decision-making authority may be uneven. For example, women are underrepresented in positions with decision-making power in the Swedish labor market, so-called *vertical gender segregation* (Keisu et al., 2021; Nordic Statistics Database, 2021). This is especially pronounced for the highest positions, and in the private sector. Sweden is ranked as one of the world’s leading countries with regard to organizational gender equality (European Institute for Gender Equality, 2023), however, only 28 percent of executives and 12 percent of CEOs in the country are women (Nordic Statistics Database, 2021), indicating that women’s underrepresentation is particularly pronounced at the top of Swedish organizations. Given that occupying decision-making positions is likely linked to increased access to decision-making authority, particularly at the organizational level, there may be gender differences in decision-making access at this level.

Some past research has found evidence for gender differences in experienced PDM (Valverde-Moreno et al., 2019, 2021a,b), but other research has found no differences (Kahnweiler and Thompson, 2000; Li and Qian, 2016). Some studies have included items related to PDM both at the workplace and organization level, however, these studies do not allow for a comparison of results of experienced PDM at both levels, as the responses were either combined into a single PDM scale (Kahnweiler and Thompson, 2000) or items that measure PDM at the organizational level were given more weight due to their greater contribution to the explained variance (European Working condition Survey; Valverde-Moreno et al., 2019, 2021a). When not giving more weight to PDM at the organizational level, but using the same data (European Working Condition Survey; Valverde-Moreno et al., 2021b), women actually experienced less organizational PDM than men, whereas surprisingly, women experienced slightly more workplace PDM than men. Also, to the best of our knowledge, the only study that has solely investigated workplace PDM did not reveal any gender differences (Li and Qian, 2016). Taken together, the observed inconsistencies regarding gender differences in experienced PDM in past research may be explained by more pronounced gender differences at the organisational level compared to the workplace level.

*Hypothesis 1*: Women experience lower PDM than men, this difference is more pronounced at the organizational level compared to the workplace level.

### Gender differences in organizational PDM- a matter of occupational roles?

Male-typed occupational roles encompass professions (e.g., electricians, engineers) and status positions (e.g., leadership, upper management. Whether an occupational role is perceived as male- or female-typed corresponds to the gender distribution commonly found in that role (Cejka and Eagly, 1999; Caleo and Heilman, 2014). Men are overrepresented in occupations perceived to require agentic traits (e.g., dominant, competitive, while women are overrepresented in occupations perceived to require communal traits (e.g., nurturing, gentle; Cejka and Eagly, 1999), so-called *horizontal gender segregation*. In Sweden, women make up only 22 percent of employees working in occupations stereotypically associated with men, while accounting for 73 percent of employees working in occupations stereotypically associated with women (Nordic Statistics Database, 2022). Furthermore, only 16 percent of women and 15 percent of men work in occupations with an equal gender distribution (Arbetsmiljöverket [Swedish Work Environment Authority], 2017). As such, in addition to the vertical gender segregation described above, horizontal gender segregation is also highly prevalent in the Swedish labor market (Keisu et al., 2021).

Both horizontal and vertical gender segregation consolidate gender stereotypic beliefs that agentic characteristics and behaviors are necessary for success in male-typed occupational roles. This continues to foster the idea that men are better suited for male-typed professions (Caleo and Heilman, 2014) and leading positions (Eagly and Karau, 2002) than women. To be granted influence in such male-typed roles, embodying agentic values and practices such as competitiveness, assertiveness, independence, and meeting agency norms is often required (Cheryan and Markus, 2020). As a result, women may experience difficulties gaining access to influence in male-typed professions and positions (Eagly and Karau, 2002; Heilman and Caleo, 2018). For instance, occupying leadership positions does not come with the same amount of influence for women as it does for men; female leaders are often given less influential tasks (Rebérioux and Roudaut, 2019) and are less likely to be asked for their opinion by their CEOs compared to male leaders (Mooney, 2022).

The challenges that women face in male-typed roles are likely to be a result of gender stereotypes and the perceived incongruity between traits typically ascribed to women and the agentic requirements of male-typed roles. As people use perceived traits of a person as a basis for performance expectations (Ridgeway and Bourg, 2004), it is likely that people will assume that women are less capable of performing well in a male-typed role. Furthermore, if women contradict social norms by exhibiting counter-stereotypical agentic behavior, they may evoke penalization and backlash (Heilman, 2012). Research suggests that in order to be granted influence, women in male-typed roles need to demonstrate both communal and agentic traits. For instance, to overcome backlash effects, women working in male-typed professions were required to exhibit agentic self-confidence as well as stereotype-congruent prosocial behaviors to gain influence in organizational decision-making; in contrast, men only had to display self-confidence to be granted influence (Guillén et al., 2018). Due to the above-described findings on masculine norms in organizations, it is reasonable to assume that the gender difference in experienced PDM at the organizational level is greater in male-typed professions and positions.

*Hypothesis 2*: Gender differences in experienced PDM at the organizational level are more pronounced in (a) male-typed compared to non-male- typed professions and (b) leadership positions compared to non-leadership positions.

## Methods

### Sample

Participants were drawn from the Swedish Longitudinal Occupational Survey of Health (SLOSH), wave 7 (conducted in 2018, *N* = 17,841). SLOSH is an open cohort survey of an approximately nationally representative sample of the working population in Sweden (for a detailed description see Magnusson Hanson et al. (2018)). Participants chose between two versions of the questionnaire, one for those currently in paid work and one for those permanently or temporarily outside the labor force. Only those who answered the questionnaire for those in paid work in 2018 were included in the analyses (*N* = 11,552). After the exclusion of self-employed participants, the study sample consisted of *N* = 10,500 individuals (60% women and 40% men, with a mean age of 52.5 (*SD* = 9.3)).

### Measures and procedure

#### PDM at the workplace and organizational level

Experienced workplace PDM was assessed with the item “How much influence do you think you have in your workplace?.” Experienced organizational PDM was assessed with the item “How much influence do you think you have in the organization/company you work in?.” Responses were indicated on a 5-point Likert scale (1 = *not at all*, 5 = *very much*).

#### Participants’ gender

Participants’ gender was indicated in the SLOSH dataset and originally obtained by linkage to the “Longitudinal integrated database for health insurance and labor market studies” (LISA) and indicated as woman (=1) or man (=0).

#### Participants’ profession

To determine the gender typicality of participants’ professions we used the three-digit level of the categorization by the Swedish Occupational Classification System (SSYK; Statistics Sweden 2012). The SSYK data on occupational gender composition in occupations in Sweden for the year 2018 were obtained from Statistics Sweden (www.scb.se). Professions were categorized as *male-typed* (<40% women, e.g., engineers, installation electricians) and *non-male-typed* (>40% women, e.g., architects, high school teachers, nurses). The chosen labels were intended to highlight the study’s focus on experiences within male-typed contexts. They also signify that the non-male-typed category includes a range of gender compositions, including gender-balanced and female-typed.

#### Participants’ position

Participants’ position was assessed with the item; “Do your responsibilities involve leading or distributing the work of others?” including four response options; “No,” “Yes, I am a supervisor but not a manager,” “Yes, I am a manager without personnel responsibility,” and “Yes, I am a manager with personnel responsibility.” For the purpose of this study, the responses were recategorized into ‘non-leadership position’ entailing the first response option, and ‘leadership position’ comprising the latter three response options.

#### Control variables

To account for possible influence, we conducted additional analyses including tenure and working time (full-time or part-time) as control variables. Adding the control variables as covariates did not impact the direction and strength of the examined relationships (for details see Appendix), we therefore did not include the control variables in the following analyses.

## Results

### Descriptives

Table 1 includes the distribution of women and men in the male-typed and non-male-typed professions and the distribution within each profession in leadership and non-leadership positions. More women than men responded to the survey, and the sample includes a substantial representation of leaders, constituting 31% of the respondents. The prevalent horizontal and vertical gender segregation of the Swedish labor market (see above) is reflected in our sample; 67% of the men in the sample were working in male-typed professions, while 87% of women worked in non-male-typed professions. Similarly, a larger proportion of men (38%) indicated occupying a leadership position compared to the proportion amongst women (26%). The representation of leaders was higher in male-typed (38%) than non-male typed positions (27%). Men were equally represented as leaders in male- (38%) and non-male professions (37%), whereas women were more often represented as leaders in male-typed (37%) than non-male-typed position (25%). As such, women in male-typed professions had a comparable likelihood of occupying a leadership position as men. However, in non-male-typed professions, men were disproportionately represented in leadership positions. Furthermore, a larger percentage of women (31%) than men (10%) was working part-time. Women, *M* = 15.67, *SD* = 12.40, and men, *M* = 15.25, *SD* = 12.23, indicated similar values on the measure of tenure (in years). The correlation of PDM at the workplace and organizational levels was, *r*(10413) = 0.64, *p* < 0.001.

**Table 1:** Number of women and men in the sample by gender typicality of profession and leadership position.

	Women		Men		Total	
	N	%	N	%	N	%
Total	5,780	60%	3,785	40%	9,565	100%
Leader	1,516	26%	1,421	38%	2,937	31%
Non-Leader	4,264	74%	2,364	62%	6,628	69%
		100%		100%		
Non-male-typed (> 40% women)	5,014	80%	1,259	20%	6,273	100%
Leader	1,232	25%	470	37%	1,702	27%
Non-Leader	3,782	75%	789	63%	4,571	73%
		100%		100%		
Male-typed (< 40% women)	766	23%	2,526	77%	3,292	100%
Leader	284	37%	951	38%	1,235	38%
Non-Leader	482	63%	1,575	62%	2,057	62%
		100%		100%		

The percentages of leaders and non-leaders refer to the percentage within each gender.

### PDM at workplace and organizational levels

**Figure 1:**
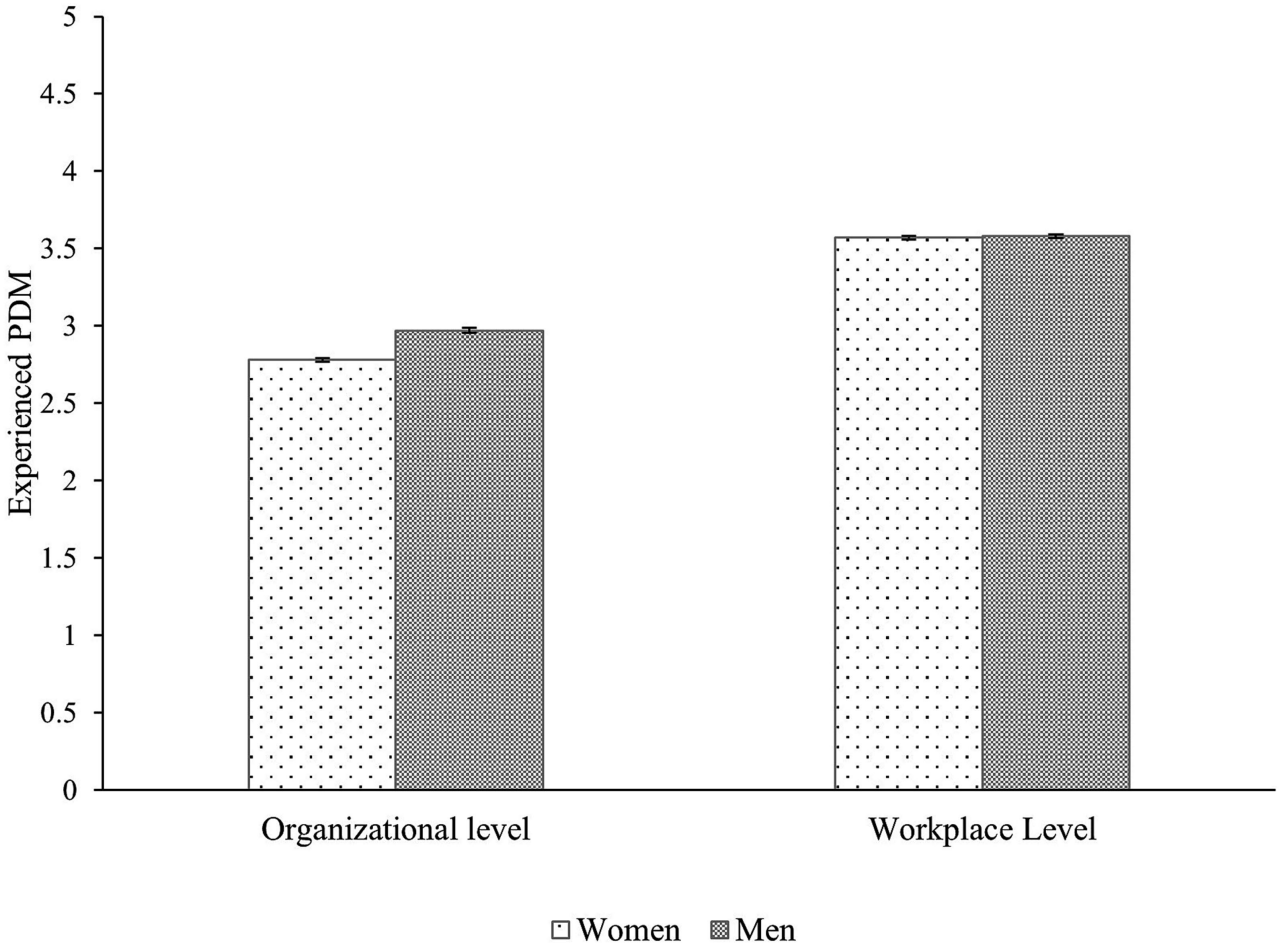
Means and standard errors of experienced PDM by participants’ gender and level of assessment.

To test whether women experience lower levels of PDM than men and whether this difference is more pronounced at the organizational level compared to the workplace level (Hypothesis 1), we conducted an ANOVA of the 2 Participants’ Gender (women, men) as between-subject variable × 2 Levels (workplace, organizational) as within-subject variable. This analysis revealed a main effect of participants’ gender, *F*(1,10413) = 33.78, *p* < 0. 001, η_p_^2^ = 0.003, with women (*M* = 3.18, *SD* = 1.12) reporting lower experienced PDM than men (*M* = 3.28, *SD* = 1.33). Moreover, the effect of level was significant, *F*(1,10413) = 7486.99, *p* < 0.001, η_p_^2^  = 0.418; indicating that participants experienced less PDM at the organizational level (*M =* 2.86, *SD* = 1.03), than at the workplace level (*M* = 3.58, *SD* = 0.85). Furthermore, the Participants’ Gender × Level interaction was significant, *F*(1,10413) = 115.53, *p* <0.001, η_p_^2^ = 0.011. Two independent t-tests revealed that at the organizational level, women (*M* = 2.78, *SD* = 1.01) experienced less PDM than men (*M* = 2.97, *SD* = 1.06; *t*(10428) = 9.05, *p* < 0.001, *d* = 0.18), whereas at the workplace level, women (*M* = 3.57, *SD* = 0.82) and men (*M* = 3.58, *SD* = 0.88) did not differ in their experienced PDM, *t*(10432) = 0.60, *p* = 0.546, *d* = 0.01. Comparisons of the means are depicted in Figure 1.

### Gender differences at the organizational level

To examine whether the gender difference in experienced PDM at the organizational level is more pronounced in male-typed positions and professions (Hypothesis 2), we conducted a 2 Participants’ Gender (women, men) × 2 Position (leader, non-leader) × 2 Profession (male-typed, non-male-typed) ANOVA. This analysis revealed a significant main effect of participants’ gender, *F*(1,9557) = 38.00, *p* < 0.001, η_p_^2^ = 0.004, and Position, *F*(1,9557) = 603.76, *p* <. 001, η_p_^2^ = 0.059. The main effect of profession was not significant, *F*(1,9557) = 0.28, *p* = 0.595, η_p_^2^ < 0.001. As shown in Table 2, depicting the means and standard deviations by gender, position, and profession, employees in leadership positions reported higher experienced PDM than employees in non-leadership positions.

**Table 2:** The number of participants, means, and standard deviations of experienced PDM at the organizational level separated by participants’ gender, leadership position, and gender typicality of profession.

		PDM at the organizational level					
		Women		Men		Total	
		M	SD	M	SD	M	SD
		2.77	1.00	2.97	1.05	2.85	1.03
Position	Leader	3.18	1.02	3.42	0.99	3.29	1.01
	Non-Leader	2.63	0.96	2.70	1.00	2.66	0.97
Profession: Male-typed (< 40% women)	Leader	3.25	1.07	3.42	0.99	3.38	1.01
	Non-Leader	2.55	1.00	2.68	1.01	2.65	1.01
	Total	2.81	1.08	2.96	1.07	2.92	1.07
Profession: Non-male-typed (> 40% women)	Leader	3.16	1.01	3.40	0.99	3.23	1.01
	Non-Leader	2.64	0.95	2.75	0.97	2.66	0.95
	Total	2.77	0.99	3.00	1.03	2.81	1.00

M, Mean; SD, Standard Deviation.

**Figure 2:**
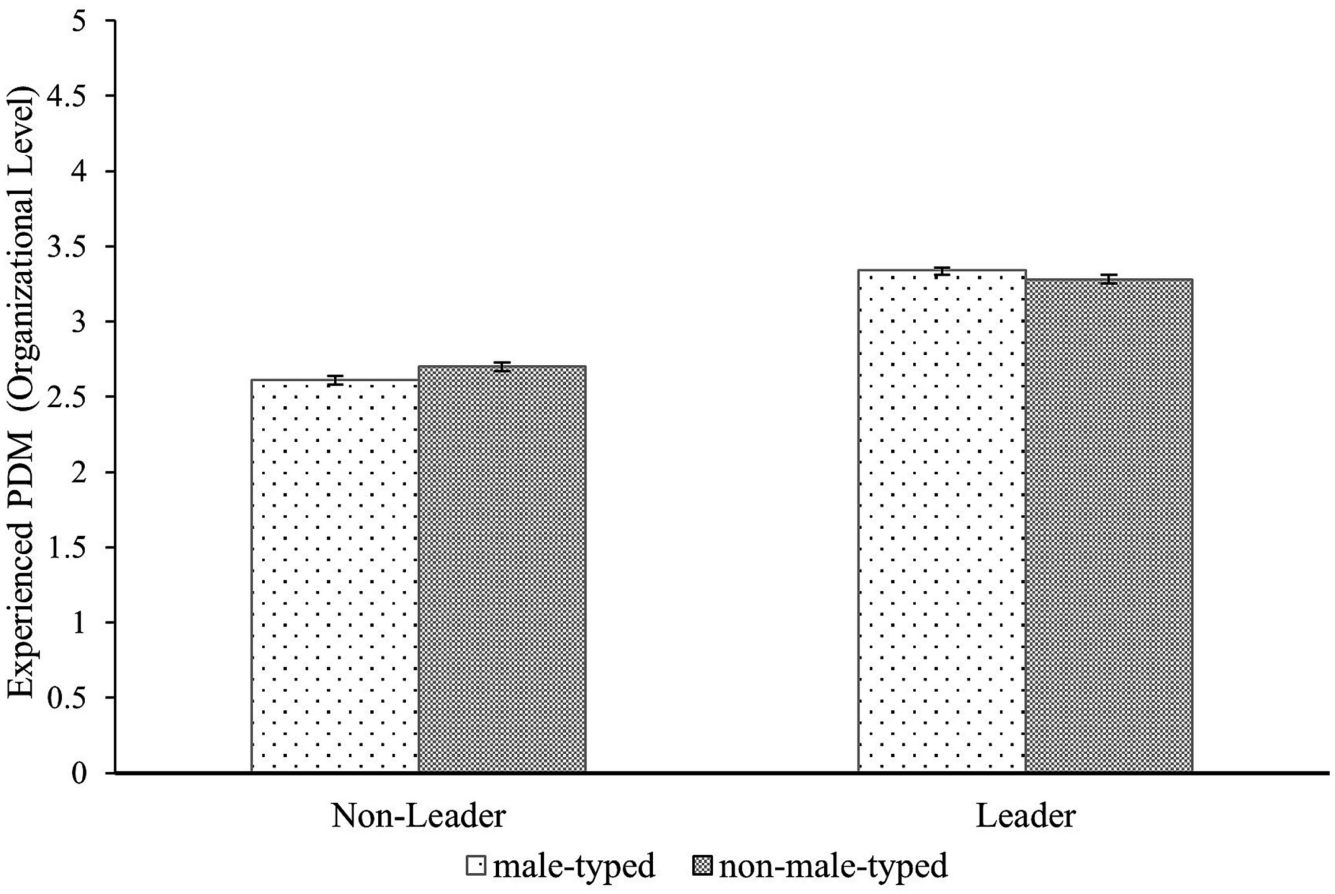
Means and standard errors of experienced PDM at the organizational level by participants’ position and gender typicality of profession.

The Participants’ Gender × Position interaction was not significant, *F*(1,9557) = 2.70, *p* = 0.100, η_p_^2^ < 0.001. Similarly, the Participants’ Gender × Profession interaction was not significant, *F*(1,9557) = 0.28, *p* = 0.596, η_p_^2^ < 0.001. Unexpectedly, the analysis revealed a significant Position × Profession Interaction, *F*(2,9557) =6.47, *p* = 0.011, η_p_^2^ = 0.001. Tukey HSD *post hoc* analysis revealed that participants occupying non-leadership positions experienced less PDM in male-typed professions (*M* = 2.61, *SD* = 1.01) compared to non-male typed professions (*M* = 2.70, *SD* = 0.95), *t*(9557) = −2.56, *p* = 0.051, *d* = −0.08. The comparison of experienced PDM of employees in leadership positions in male-typed professions (*M* = 3.34, *SD* = 1.01) compared to non-male-typed professions (*M* = 3.28, *SD* = 1.01) was not significant t(9557) = 1.26, *p* = 0.589, *d* = 0.05. The comparison of means is depicted in Figure 2.

The three-way interaction between Participants’ Gender × Position × Profession was not significant, *F*(2,9557) = 0.563, *p* = 0.453, η_p_^2^ < 0.001. As such, the results do not lend support to the assumption that gender differences in experienced PDM at the organizational level are amplified in male-typed positions and professions.

## Discussion

The purpose of this study was to investigate women’s and men’s experiences of PDM, differentiating between levels of decision-making, and gender typicality of the work context. Specifically, we examined whether women perceive less PDM than men and whether this difference is more pronounced at the organizational level compared to the workplace level. Further, we examined whether the gender difference at the organizational level is more pronounced among employees in male-typed positions and professions than in other contexts. By differentiating between the organizational and workplace level in the assessment of PDM and taking the gender-typicality of the context into account, the present study sheds light on previous inconclusive results regarding gender differences in PDM. The results demonstrate that women experienced less PDM than men at the organizational level, whereas they did not differ in their experiences of PDM at the workplace level. Previous assessments of PDM at only one level, or a combination of both workplace and organizational levels, may have contributed to the inconsistent findings regarding gender differences in PDM. Our results are in line with previous findings using self-reported PDM, showing that women reported less organizational PDM than men (Valverde-Moreno et al., 2021b). We theorize that the finding of gender differences in PDM at the organizational level reflects the idea that male-stereotypical traits are more relevant for decision-making with wide-ranging implications (Huddy and Terkildsen, 1993), resulting in women being granted and thus also experiencing less influence in organizational decision-making.

We did not find more pronounced gender differences in male-typed compared to non-male-typed positions and professions. Women were not particularly negatively affected by stereotypes or experienced less influence in decision-making in male-typed occupational roles. As such, there may be factors other than gender typicality that influence women’s and men’s perception of inclusion in decision-making. The data revealed an additional noteworthy pattern about the relative frequency of leaders within one gender. In male-typed professions, the proportion of female (37%) and male leaders (38%) were similar. While this might reflect an overrepresentation of women in leadership roles within male-dominated professions in our sample, additional explanations are also possible. The observed pattern could reflect a positive selection process, wherein women who persevere in male-dominated contexts are more likely to progress to leadership positions (di Paola et al., 2022). Additionally, male-dominated fields (e.g., IT-industries) have often implemented initiatives aimed at encouraging women to enter these professions (Tokbaeva and Achtenhagen, 2023). There was no indication of the gender difference in PDM at the organizational level being dependent on the profession (male-typed vs. non-male-typed) which may reflect the success of such gender equality strategies. Comparisons of occupations with varying gender ratios in Sweden indicated that both women and men were more likely to report low decision authority in social occupations (e.g., education, health, and social work) compared to knowledge-intensive occupations (e.g., financial, insurance, technical activities; Cerdas et al., 2019; Nyberg et al., 2021). Based on these results, it appears that additional characteristics of professions, such as decision-making structures, must be considered to evaluate the relationship between work context, gender, and inclusion in decision-making.

### Methodological considerations and future research directions

The present study has certain limitations and raises questions that future research should address. The effect sizes associated with the observed gender differences are notably small. Several factors potentially contribute to these rather small effect sizes, such as their reflection of Sweden as a relatively gender-equal context or potential gender differences in expectations regarding the extent of influence granted in organizations. It is, however, essential to recognize that even slight disparities can have meaningful consequences overall, especially when considering the cumulative impact over time and across various organizational contexts. The persistence of gender differences in perceived participative decision-making warrants attention, even in the face of small effect sizes. The importance of such differences lies not only in their immediate impact but also in their potential to perpetuate broader gender inequalities, for instance, in job satisfaction (Pacheco and Webber, 2016) and career progression (Nyberg et al., 2015).

The measures we used to assess PDM have shortcomings, primarily resulting from the use of existing data; we utilized single items to assess experienced PDM at workplace and organizational levels. Detailed aspects of the concept of PDM, such as further details of the type of decision-making, were thus not accounted for by the used measure. Furthermore, this approach raises questions about the distinction between perceived influence, as captured by our study, and actual participatory decision-making opportunities. There might also be variability in gender differences in terms of the amount of influence they anticipate, affecting how they perceive and rate the extent of their influence. Therefore, future research should utilize more multifaceted measures of PDM and include assessments of opportunities for influence and actual participatory decision making (on strategic or operational matters). Furthermore, the design of the study was cross-sectional and thus could only reveal associations between employees’ position, profession, and PDM. Further studies are needed to approximate the causal effect of gender stereotypes and individual and organizational attributes. Although the study was distributed to a nationally representative sample, variations in response rates may pose limitations to the generalizability of the study. For instance, leaders and women are overrepresented. This overrepresentation is probably associated with an overrepresentation of highly-educated respondents (response rate: post-secondary education = 55%, pre-secondary education = 42%), as individuals with advanced degrees are more likely to be hired or promoted to leadership positions. The overrepresentation of women is likely linked to a higher propensity for women to participate in surveys. However, there is an observed underrepresentation of women in male-typed professions, compared to other measures of gender distribution in such professions (Nordic Statistics Database, 2022). This observation may again reflect disparities in response rates related to education, given that many female-typed occupations, like teaching and nursing, often demand post-secondary education. Consequently, our results may present an incomplete representation of the experiences of employees without leadership responsibility and women in male-typed professions and cannot be generalized to individuals with pre-secondary education in the Swedish labor market. Considering the association between leadership positions and participatory decision-making (PDM), our findings might also overemphasize the perceived levels of PDM. Moreover, it is important to acknowledge that the generalizability of our study findings may be limited due to the use of data exclusively from Sweden. Cross-cultural studies indicate that cultural values (e.g., masculinity, power distance; Valverde-Moreno et al., 2019) are related to the degrees of observed PDM and that approaches to PDM can vary significantly across different cultures (Sagie and Aycan, 2003). Future research thus needs to examine whether these findings can be replicated in other countries.

In addition to the different levels where decisions are made, involving employees in PDM also entails employing different approaches to share authority and power. This includes consultation, where input is sought from employees, and delegation, which grants employees independent decision control (Richardson et al., 2021; Lundmark, 2023). Research indicates that the method of authority sharing has varying effects on employee related outcomes at different levels of decision-making (Richardson et al., 2021). To enhance our understanding of the interplay of power/authority sharing, and gender on PDM, future studies should investigate whether gender differences exist in the types of authority given to men and women. As such, male employees might more frequently encounter delegation, while female employees might be primarily consulted, leading to disparities in the recognition they receive for their contributions, perceived levels of PDM and influence on career progression. The exploration of such differences should consider potential variations across decision-making levels and examine how these differences are linked to the perceptions of inclusion in decision-making processes for both women and men.

The finding that leadership positions were associated with a greater perception of PDM at the organizational level emphasizes potential consequences of the vertical gender segregation in the Swedish labor market, where women overall hold fewer leadership positions compared to men. The underrepresentation of women in leadership positions might be a factor in explaining gender differences in PDM at the organizational level. As reflected in our dataset, women are more likely to hold part-time positions compared to men (Statistics Sweden, 2022). Additionally, women spend more time on unpaid domestic work compared to men (Statistics Sweden, 2021). Both part-time employment and work/life conflict relate to vertical gender segregation as they inhibit career progress (e.g., by inhibiting building networks), and work/life conflict also leads to exiting leadership positions among women (Eagly and Carli, 2018). As such, these other aspects likely contribute to women being underrepresented in leadership positions and experiencing less involvement in decision-making at the organizational level.

Despite the acknowledged limitations, it is important to note the strengths of our study, these include the use of an approximately representative sample of the Swedish workforce, as well as its large sample size, which allows the detection of even small effects. Our results are consistent with previous findings that women are less likely to be included in organizational decision-making than men (Mooney, 2022) and indicate that women’s lack of inclusion at the organizational level is reflected in their perceptions and experience of PDM. It is employees’ perception of PDM that has been found to be associated with positive employee outcomes such as job satisfaction. As a result, women may be less likely to benefit from the positive outcomes of PDM compared to men. More research is needed on how women’s experience of PDM affects work-related outcomes and their careers.

### Implications for Practice

Organizational gender diversity has been related to improved quality of decision-making as a consequence of the larger variety of perspectives considered (Larrieta-Rubín de Celis et al., 2015). Current gender equality efforts (e.g., gender quotas) largely focus on the distribution of men and women in leadership positions. However, our findings demonstrate that women—regardless of their position—perceive having less influence on decision-making at the organizational level than men. This indicates that equal gender distribution in high-level positions is not synonymous with an equal distribution of power and does not necessarily mean that all perspectives are considered equally (Mooney, 2022). To achieve gender equality in organizations, practitioners should take PDM into account and facilitate opportunities for women to influence organizational decision-making.

## Conclusion

It is possible that the inconclusive findings of previous research examining gender differences in PDM relate to the level at which PDM was assessed. Based on the results of this study, women experienced less PDM than men at the organizational level, but not at the workplace level. Therefore, when evaluating gender equality at work and developing gender equality strategies, it is important to consider the inclusion of women and men in strategic, large-scale decisions.

## Data availability statement

The data analyzed in this study is subject to the following licenses/restrictions: Given restrictions from the ethical review board and considering that sensitive personal data are handled, it is not possible to make the data freely available. Access to the data may be provided to other researchers in line with Swedish law and after consultation with the Stockholm University legal department. Requests for data, stored at the Stress Research Institute, Department of Psychology, should be sent to registrator@su.se with reference to GENDER DIFFERENCES IN EXPERIENCED PDM (2022–02) or directly to the corresponding author. Requests to access these datasets should be directed to registrator@su.se.

## Ethics statement

The studies involving humans were approved by the Regional Research Ethics Board in Stockholm. The studies were conducted in accordance with the local legislation and institutional requirements. The participants provided their written informed consent to participate in this study.

## Author contributions

CP, MG, CB-O, CL, and SS contributed to conception and design of the study. CL organized the database. CP performed the statistical analysis. CP has been responsible for drafting the manuscript and revising with the contribution of all co-authors.

## Appendix

### Analysis controlling for tenure and working time

#### PDM at Workplace and Organizational Levels

A 2 Participants’ Gender (women, men) × 2 Levels (workplace, organizational) ANCOVA was conducted to control for the impact of the variables tenure and working time. The analysis revealed a main effect of both tenure, *F*(1,8769) = 29.53, *p* <0.001, η_p_^2^ = 0.003, and working time, *F*(1,8769) = 74.30, *p* < 0.001, η_p_^2^ = 0.008. The main effect of participants gender, *F*(1,8769) = 10.33, *p* = 0.001, η_p_^2^ = 0.001, and the effect of level, *F*(1,8769) = 2150.62, *p* < 0.001, η_p_^2^ = 0.197, remained significant. Furthermore, the Participants’ Gender × Level interaction remained significant, *F*(1,8769) = 91.55, *p* < 0.001, η_p_^2^ = 0.010.

#### Gender Differences at the Organizational Level

A 2 Participants’ Gender (women, men) × 2 Position (leader, non-leader) × 2 Profession (male-typed, non-male-typed) ANCOVA was performed to address the potential impact of the control variables tenure and working time. The effects of both tenure, *F*(1,8167) = 13.37, *p* < 0.001, η_p_^2^ = 0.002, and working time, *F*(1,8167) = 31.08, *p* < 0.001, η_p_^2^= 0.004, were significant. In accordance with the previous analysis, the analysis including the control variables revealed significant main effects of participants’ gender, *F*(1,8167) = 31.29, *p* < 0.001, η_p_^2^ = 0.004, and position, *F*(1,8167) = 506.72, *p* < 0.001, η_p_^2^ = 0.058. Again, no evidence was found for a main effect of profession, *F*(1,8167) = 1.23, *p* = 0.268, η_p_^2^ < 0.001, a two-way interaction of Participants’ Gender × Position, *F*(1,8167) = 3.67, *p* = 0.055, η_p_^2^ < 0.001, or a two-way interaction of Participants’ Gender × Profession, *F*(1,8167) = 0.11, *p* = 0.744, η_p_^2^ < 0.001. As before, a significant interaction was found between Position × Profession, *F*(1,8167) = 7.64, *p* = 0.006, η_p_^2^ = 0.001, and the three-way interaction Participants’ Gender × Position × Profession remained non-significant, *F*(1,8167) = 0.69, *p* = 0.405, η_p_^2^ < 0.001.
